# Applicability of Retzius striae in forensic anthropology: a systematic review

**DOI:** 10.1007/s00414-026-03954-3

**Published:** 2026-08-13

**Authors:** Clarice de Araújo Oliveira, Antônio do Rêgo Castelo-Branco, Paulo Henrique Viana Pinto, Ricardo Henrique Alves da Silva

**Affiliations:** 1 Department of Pathology and Legal Medicine, Ribeirão Preto Medical School, University of São Paulo (USP), Ribeirão Preto, São Paulo Brazil; 2 Forensic Odontologist, Bahia State Scientific Police, Bahia Camaçari, Brazil; 3 Department of Stomatology, Public Health, and Forensic Odontology, Ribeirão Preto School of Dentistry, University of São Paulo (USP), Avenida do Café, s/n, Ribeirão Preto, São Paulo 14040-904 Brazil

**Keywords:** Forensic anthropology, Forensic dentistry, Tooth, Dental enamel, Biomarkers, Retzius striae

## Abstract

Forensic Anthropology applies biological characteristics in legal contexts to estimate the biological profile of an unknown individual. In this regard, teeth stand out due to their resistance and ability to preserve valuable information over time. More specifically, microstructural records present in dental enamel, in the form of incremental markings known as Retzius striae, provide data on dental development and, consequently, on the individual. The objective of this systematic review was to assess the applicability of Retzius striae in Forensic Anthropology. A search was conducted using topic-related keywords across six different databases in Portuguese, Spanish, and English. Studies were selected according to predefined inclusion and exclusion criteria. The methodological quality and risk of bias of the included studies were assessed. As a result, seven scientific studies addressing Retzius striae in contemporary human samples were identified. All investigations evaluated the striae using histological methods. Furthermore, all studies demonstrated a low risk of bias. Retzius striae have been investigated as markers for estimating age, sex, stature, and even body weight; however, the available evidence remains limited. Although they show potential, all these applications require further validation. Therefore, additional standardized and representative primary studies are necessary to ensure reliable and safe application in the forensic context.

**Supplementary Information:**

The online version contains supplementary material available at https://doi.org/10.1007/s00414-026-03954-3.

## Introduction

Forensic Anthropology analyzes human remains in legal contexts to estimate the biological profile of an unknown individual which corresponds to a set of biological characteristics that can be estimated postmortem and are capable of describing the individual during life, particularly at the time of death [1]. Traditionally, it encompasses the estimation of sex, age at death, population affinity, and stature, with the purpose of narrowing a list of missing persons and, consequently, assisting in identification [1, 2].

Within this framework, the skeleton represents a record of an individual’s life experiences, including living conditions, habits, health status, and traumatic events [1]. Among the structures that compose this biological framework, teeth stand out for their hardness and capacity to preserve valuable information over time [3], given their lower susceptibility to postmortem alterations resulting from taphonomic processes when compared to other morphological references, such as bones [4, 5].

In forensic practice, teeth exhibit unique characteristics that can be utilized to establish a biological profile [6], as they retain a record of their own growth and development, providing valuable insights into an individual’s life history. This information is preserved within the three mineralized dental tissues (enamel, dentin, and cementum) [7] and is typically expressed through internal macro- and microstructural features. Such features can be systematically examined, particularly through histological techniques [8], to assess and reconstruct the biological profile of individuals.

Among these characteristics, the microstructural records present in dental enamel stand out, such as incremental marks, which provide a permanent record of enamel formation. Physiological incremental markings include cross-striations, which reflect the daily secretory rhythm of ameloblasts [8], and Retzius striae (also referred to as Retzius lines), which represent a longer-period endogenous rhythm of enamel deposition [9]. In contrast, accentuated incremental markings, such as the neonatal line and Wilson bands, are episodic structures formed in response to disruptions of amelogenesis [10, 11]. The neonatal line represents a pronounced accentuated stria associated with the physiological stress of birth [10], whereas Wilson bands are related to other episodes of systemic or local stress affecting enamel formation [11]. Together, these enamel records provide valuable information on dental development, growth patterns, developmental timing, and episodes of physiological stress throughout an individual’s life history [12, 13].

Retzius striae were first described in the study conducted by Anders Adolf Retzius in 1837 as “brownish parallel lines” [9]. Upon examination of the internal structure of teeth, parallel lines were identified in the enamel and interpreted as traces of different periods of enamel formation, that is, periodic markings reflecting the history of tooth development [9]. This periodic pattern of enamel formation is characteristic of mammals [14].

These striae are incorporated into the developing enamel as enamel-forming cells, namely ameloblasts, deposit new matrix [8, 13]. Consequently, when a tooth is sectioned and examined under a microscope, Retzius striae become visible as regular dark lines [8]. On the enamel surface, Retzius striae are externally expressed as perikymata, which, unless worn or damaged, are visible as a series of horizontal grooves and ridges covering the crown surface [15, 16].

Retzius periodicity, also referred to as the long-period repeat interval, corresponds to the number of daily enamel increments (cross-striations) between two adjacent Retzius lines and is determined by counting these increments [8, 13, 17, 18]. In humans, this periodicity ranges from 5 to 12 days [8, 12, 13, 16–19] and constitutes a fundamental parameter for more accurately estimating crown formation time, enabling developmental events to be placed within a temporal framework of days and weeks rather than only months and years [12].

The etiology of Retzius periodicity in humans has been extensively investigated; however, it has not yet been conclusively established [8, 13]. It is believed to reflect an underlying infradian biological rhythm regulated by an internal biological clock [8, 13, 14, 20, 21]. Within the framework of chronobiology, these rhythms extend beyond 24 h and differ from circadian rhythms, which govern daily physiological cycles [17, 22]. Evidence suggests that such rhythms are closely associated with various aspects of human biology, including tissue and organ development and maintenance, as well as body mass regulation [7, 14, 22].

Several hypotheses have been proposed to explain the origin of this rhythm. Early studies suggested that Retzius periodicity (RP) might arise from the interaction of two imperfectly synchronized circadian rhythms [8, 23]. Another hypothesis identifies the hypothalamic suprachiasmatic nucleus as a central regulator, proposing that rhythmic modulation of autonomic nervous system activity and cardiovascular function could generate the periodic metabolic fluctuations recorded as Retzius striae [7, 8, 13, 14, 24]. Expanding upon this concept, the Havers–Halberg Oscillation hypothesis proposes that centrally regulated hypothalamic and endocrine oscillations coordinate long-period biological rhythms associated with skeletal growth, linking Retzius periodicity to both bone lamellar formation and body mass [7, 8, 14]. However, recent evidence indicates that RP may vary among different teeth within the same individual, suggesting that a single underlying oscillator is unlikely to fully account for its expression and that local genetic or hormonal regulatory mechanisms may also contribute to determining Retzius periodicity [8, 21, 25].

Accordingly, Retzius striae constitute a tool for understanding the timing, formation, and variability of human dental development and, indirectly, aspects related to an individual’s metabolism and physiology, thereby highlighting their potential in biological research. Nonetheless, although their existence and relevance have been discussed in the scientific literature, their applications in the field of Forensic Anthropology have not yet been fully explored. This scenario reveals a scientific gap that justifies the conduct of a systematic review aimed at identifying the potential applications of Retzius striae in Forensic Anthropology.

## Materials and methods

Initially, the PICO strategy (P – Population; I – Intervention; C – Comparison; O – Outcome) was employed to guide the formulation of the research question for this systematic review. Thus, eligible studies were defined as investigations based on data derived from contemporary human subjects (P) that included the assessment of Retzius striae (I), in which the biological characteristics of the participants were known (C), and in which the evaluation of Retzius striae could indicate applicability in Forensic Anthropology (O). Thus, the present study was developed based on the following guiding question: What are the applications of Retzius striae in Forensic Anthropology?

Therefore, the Preferred Reporting Items for Systematic Reviews and Meta-Analyses (PRISMA) protocol, 2020 version, was adopted [26]. The systematic review was registered in the International Prospective Register of Systematic Reviews (PROSPERO) under registration number CRD420250652032.

### Search strategy for obtaining studies

The search for studies was conducted in the PubMed/MEDLINE (https://pubmed.ncbi.nlm.nih.gov/), Embase (https://www.embase.com/search/quick), Scopus (https://www.scopus.com/search/form.uri?display=basic#basic), Web of Science (https://www.webofscience.com/wos/woscc/smart-search), LILACS (https://lilacs.bvsalud.org/), and SciELO (https://www.scielo.org/) databases on February 10, 2025. The Boolean operators “OR” and “AND” were used to combine the following keywords: “Forensic Anthropology”, “Forensic Odontology”, “Forensic Dentistry”, “Striae of Retzius”, “Retzius periodicity”, “Perikymata” and “Enamel formation”.

These keywords were derived from the Descriptors in Health Sciences (DeCS), Medical Subject Headings (MeSH), and Embase Subject Headings (Emtree). In addition, terms with similar meanings were employed to enhance search sensitivity. Accordingly, both the keywords and their respective synonyms, in Portuguese, English, and Spanish, were used to construct the search equations. Table 1 presents the search strategies developed and applied for each database, as well as the corresponding number of studies retrieved.

**Table 1:** Results of the equations used according to each database and language searched

Database	Search equations used according to each language		
	English	Portuguese	Spanish
PubMed/MEDLINE	((((((“Forensic Anthropology”) AND (“Forensic Dentistry”)) OR (“Forensic Odontology”)) AND (“Striae of Retzius”)) OR (“Retzius Periodicity”)) OR (“Perikymata”)) OR (“Enamel Formation”)	((((((“Antropologia Forense”) AND (“Odontologia Legal”)) OR (“Odontologia Forense”)) AND (“estria de Retzius”)) OR (“periodicidade de Retzius”)) OR (“Periquimata”)) OR (“formação do esmalte”)	((((((“Antropología Forense”) AND (“Odontología Forense”)) OR (“Odontología Legal”)) OR (“Estrías de Retzius”)) OR (“Periodicidad de Retzius”)) OR (“Periquimata”)) OR (“Formación del Esmalte”)
Result	1,086	0	0
Embase	(‘forensic anthropology’ AND ‘forensic dentistry’ OR ‘forensic odontology’) AND ‘striae of retzius’ OR ‘retzius periodicity’ OR ‘perikymata’ OR ‘enamel formation’	(‘antropologia forense’ AND ‘odontologia legal’ OR ‘odontologia forense’) AND ‘estria de retzius’ OR ‘periodicidade de retzius’ OR ‘periquimata’ OR ‘formação do esmalte’	(‘antropología forense’ AND ‘odontología forense’ OR ‘odontología legal’) AND ‘estrías de retzius’ OR ‘periodicidad de retzius’ OR ‘periquimata’ OR ‘formación del esmalte’)
Result	1,187	0	0
Scopus	( ALL ( “Forensic Anthropology” ) AND ALL ( “Forensic Dentistry” ) OR ALL ( “Forensic Odontology” ) AND ALL ( “Striae of Retzius” ) OR ALL ( “Retzius Periodicity” ) OR ALL ( “Perikymata” ) OR ALL ( “Enamel Formation” ) )	( ALL ( “Antropologia Forense” ) AND ALL ( “Odontologia Legal” ) OR ALL ( “Odontologia Forense” ) AND ALL ( “Estria de Retzius” ) OR ALL ( “Periodicidade de Retzius” ) OR ALL ( “Periquimata” ) OR ALL ( “Formação do esmalte” ) )	( ALL ( “Antropología Forense” ) AND ALL ( “Odontología Forense” ) OR ALL ( “Odontología Legal” ) AND ALL ( “Estrías de Retzius” ) OR ALL ( “Periodicidad de Retzius” ) OR ALL ( “Periquimata” ) OR ALL ( “Formación del Esmalte” ) )
Result	24	0	0
Web of science	ALL=(“Forensic Anthropology” ) AND ALL=(“Forensic Dentistry” ) OR ALL=(“Forensic Odontology” ) AND ALL=(“Striae of Retzius” ) OR ALL=(“Retzius Periodicity” ) OR ALL=(“Perikymata” ) OR ALL=(“Enamel Formation”)	ALL=(“Antropologia Forense” ) AND ALL=(“Odontologia Legal” ) OR ALL=(“Odontologia Forense” ) AND ALL=(“estria de Retzius” ) OR ALL=(“periodicidade de Retzius”) OR ALL=(“Periquimata” ) OR ALL=(“formação do esmalte”)	ALL=(“Antropología Forense”) AND ALL=(“Odontología Forense”) OR ALL=(“Odontología Legal”) AND ALL=(“Estrías de Retzius”) OR ALL=(“Periodicidad de Retzius”) OR ALL=(“Periquimata”) OR ALL=(“Formación del Esmalte”)
Result	1,377	0	0
LILACS	(“Forensic Anthropology” ) AND (“Forensic Dentistry” ) OR (“Forensic Odontology” ) AND (“Striae of Retzius” ) OR ( “Retzius Periodicity” ) OR (“Perikymata” ) OR (“Enamel Formation”)	(“antropologia Forense” ) AND (“odontologia Legal” ) OR (“odontologia Forense” ) AND (“estria de Retzius” ) OR (“periodicidade de Retzius” ) OR (“periquimata” ) OR (“formação do esmalte”)	(“Antropología Forense”) AND (“Odontología Forense”) OR (“Odontología Legal”) AND (“Estrías de Retzius”) OR (“Periodicidad de Retzius”) OR (“Periquimata”) OR (“Formación del Esmalte”)
Result	143	579	0
SciELO	(“Forensic Anthropology” ) AND (“Forensic Dentistry” ) OR (“Forensic Odontology” ) AND (“Striae of Retzius” ) OR (“Retzius Periodicity” ) OR (“Perikymata” ) OR (“Enamel Formation”)	(“Antropologia Forense” ) AND (“Odontologia Legal” ) OR (“Odontologia Forense” ) AND (“Estria de Retzius” ) OR (“Periodicidade de Retzius” ) OR (“Periquimata” ) OR ( “Formação do esmalte”)	(“Antropología Forense”) AND (“Odontología Forense”) OR (“Odontología Legal”) AND (“Estrías de Retzius”) OR (“Periodicidad de Retzius”) OR (“Periquimata”) OR (“Formación del Esmalte”)
Result	3	22	0

### Study eligibility criteria

Only cross-sectional observational studies using samples from contemporary human subjects were included, excluding research involving ancient populations, fossils, or archaeological remains. This methodological choice was adopted to ensure that the findings reflect current conditions and contexts, avoiding the inclusion of research based on ancient human remains or archaeological data, which could introduce differences of a historical or evolutionary nature. Therefore, the conclusions presented herein should be interpreted in light of evidence derived exclusively from contemporary human populations.

Furthermore, eligible studies indicated a potential direct or indirect application of Retzius striae in Forensic Anthropology. The included investigations assessed both permanent and deciduous teeth. No restrictions were applied regarding language, year of publication, or population origin.

Studies that evaluated neonatal lines, a type of Retzius line formed at the time of birth, were excluded. In addition, investigations assessing teeth with abnormalities, literature reviews, and conference abstracts were excluded, as were those that performed age estimation based on artificial intelligence, case reports, and studies involving non-human animals.

### Selection of studies by databases

Using the search equations in the databases on February 10, 2025, the retrieved records were subsequently imported into the Rayyan application (https://www.rayyan.ai/). This process enabled duplicate removal, followed by study selection based on titles and abstracts, respectively. Screening was conducted independently by two reviewers, and any discrepancies were resolved through discussion in a meeting, during which the inclusion or exclusion of each study was individually determined.

Subsequently, both reviewers proceeded with full-text reading of the studies selected in the previous stage, followed by a new round of article inclusion and exclusion. Thereafter, a comparison of the selected studies was performed, and any differences between the reviewers were clarified. The stages corresponding to study eligibility were conducted in accordance with the previously established inclusion and exclusion criteria.

### Selection of studies through manual search

After the selection of studies retrieved from the databases, a complementary manual search was conducted on February 15, 2025, in which all publications citing the previously selected studies were examined in order to identify additional relevant articles based on their titles. Subsequently, studies selected by title were assessed through abstract screening and full-text reading. Inclusion or exclusion was determined according to the previously described eligibility criteria. This stage was also performed independently by two reviewers, and any disagreements were resolved through joint discussion in a meeting.

### Data extraction from the studies

After completion of the study selection stage, data extraction was performed. For this purpose, two reviewers once again independently conducted full-text reading of each study. The extracted data were evaluated, and any discrepancies were resolved through re-examination of each study.

Subsequently, the data were entered into spreadsheets using Microsoft Excel© (Microsoft Corp., Redmond, WA, USA) and categorized according to the study’s bibliographic information (authors, year of publication, study title, journal name) and methodological information (sample size, sample origin, tooth assessed, method employed, and potential application of Retzius striae). Data related to the processing and analysis techniques of Retzius striae were also extracted, including sample preparation, microscopy technique, area of the tooth analyzed, and method used to measure Retzius periodicity. In addition, data regarding the periodicity values, expressed in days, assessed in each study were collected (tooth, sex, minimum, mean, maximum, and mode).

### Methodological quality and risk of bias assessment of the studies

To assess the methodological quality and risk of bias of the selected studies, the Checklist for Analytical Cross-Sectional Studies proposed by the Joanna Briggs Institute (JBI) [27] was used, and this stage was also carried out independently by two reviewers. The checklist consists of eight questions, each of which must be answered as “yes,” “no,” “not applicable,” or “unclear.”

Accordingly, the methodological quality of each study was judged based on the proportion of “yes” responses, expressed as a percentage. Questions answered as not applicable (NA) were not included in the calculation of the relative percentage of positive responses. Consequently, studies yielding a percentage equal to or below 49% were considered to present a high risk of bias; those with percentages between 50% and 69% were classified as having a moderate risk of bias; and studies with percentages equal to or above 70% were regarded as presenting a low risk of bias.

The questions included in the Checklist for Analytical Cross-Sectional Studies proposed by the JBI [27] are as follows: (1) Were the criteria for inclusion in the sample clearly defined? (2) Were the study subjects and the setting described in detail? (3) Was the exposure measured in a valid and reliable way? (4) Were objective, standard criteria used for measurement of the condition? (5) Were confounding factors identified? (6) Were strategies to deal with confounding factors stated? (7) Were the outcomes measured in a valid and reliable way? (8) Was appropriate statistical analysis used?

## Results

### Selection of studies by databases

**Fig. 1:**
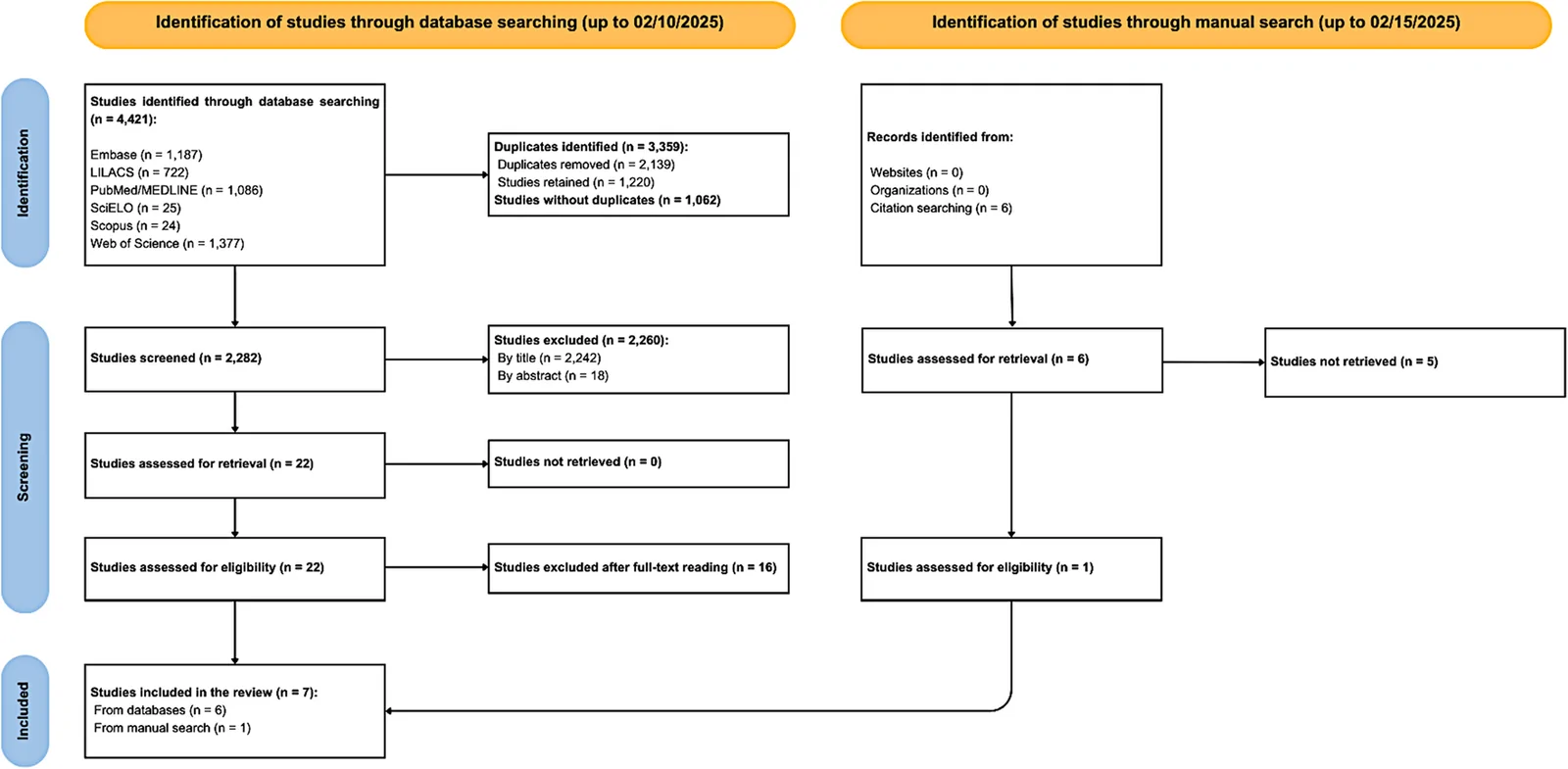
Flowchart of the study selection process. Adapted according to the PRISMA recommendations – Preferred Reporting Items for Systematic Reviews and Meta-Analyses, 2020 version [26]

The insertion of the search equations into each database enabled the retrieval of a total of 4,421 studies. The records were imported into the Rayyan application, which automatically identified 3,359 potentially duplicate records. After manual verification, 2,139 duplicate records were removed, whereas 1,220 records were retained because they were not considered true duplicates. Combined with the 1,062 non-duplicate records, a total of 2,282 studies proceeded to title screening. At this stage, 2,242 studies were excluded based on title, leaving 40 studies for abstract screening. After abstract assessment, 18 studies were excluded, and 22 studies underwent full-text evaluation. Finally, according to the predefined inclusion and exclusion criteria, six studies identified through database searching were included in this systematic review (Fig. 1).

### Selection of studies through manual search

The manual search enabled the identification of a total of six studies based on title screening. Subsequently, abstract screening was performed, resulting in the retention of two studies; during full-text assessment, one study was excluded. Finally, considering the established inclusion and exclusion criteria, one study was selected for inclusion in this systematic review together with the six studies identified in the previous stage, resulting in a total of seven included studies (Fig. 1).

### Study characteristics

The selected studies were re-read to extract bibliographic and methodological information. Table 2 presents the bibliographic details of each study, including authors, year of publication, study title, and the journal in which the study was published. The oldest study included in this review on Retzius striae was published by Reid and Dean (2006) [12], and the most recent study was by Mahoney et al. (2022) [17]. In addition, it was noted that a total of six different scientific journals were responsible for publishing the seven studies included in this systematic review. Two articles were published in the American Journal of Physical Anthropology, which changed its title to American Journal of Biological Anthropolog*y* in 2022.

**Table 2:** Bibliographic information of each included study

Authors	Year of publication	Study title	Journal name
Karaaslan et al. [19]	2021	Enamel multidien biological timing and body size variability among individuals of Chinese Han and Tibetan origins	Annals of Human Biology
Mahoney et al. [13]	2020	A structural biorhythm related to human sexual dimorphism	Journal of Structural Biology
Mahoney et al. [17]	2022	Dental biorhythm is associated with adolescent weight gain	Communications Medicine
McFarlane et al. [8]	2021	An inconstant biorhythm: The changing pace of Retzius periodicity in human permanent teeth	American Journal of Physical Anthropology
McFarlane, Littleton and Floyd [16]	2014	Estimating Striae of Retzius Periodicity Nondestructively Using Partial Counts of Perikymata	American Journal of Physical Anthropology
Reid and Dean [12]	2006	Variation in modern human enamel formation times	Journal of Human Evolution
Tan, Sim and Hsu [18]	2017	Difference in Striae Periodicity of Heilongjiang and Singaporean Chinese Teeth	Frontiers in Physiology

Regarding the methodological information of each study, it was observed that the minimum number of teeth assessed (*n* = 11) in the study by McFarlane, Littleton, and Floyd (2014) [16]. In contrast, the study by Reid and Dean (2006) [12] evaluated the largest number of teeth (*n* = 596). Furthermore, the studies analyzed incisors, canines, premolars, and molars. All selected studies employed histological assessments to obtain the data. The applications of Retzius striae addressed in the included studies, within the context of Forensic Anthropology, encompassed the estimation of age, sex, and stature (Table 3).

**Table 3:** Methodological information of each included study

Authors	Year of publication	Sample size	Sample origin	Tooth assessed	Method used	Potential application of Retzius striae
Karaaslan et al. [19]	2021	565	Beijing and Lhasa	Permanent Teeth	Histological	Stature Estimation
Mahoney et al. [13]	2020	94	Northern Great Britain	Third Molars	Histological	Sex Estimation
Mahoney et al. [17]	2022	61	New Zealand	Deciduous Molars	Histological	Stature and Sex Estimation
McFarlane et al. [8]	2021	223	United Kingdom and South Africa	Incisor, Canine, Premolar, and Molar	Histological	Age Estimation
McFarlane, Littleton and Floyd [16]	2014	11	New Zealand	Permanent Mandibular Canines	Histological	Age Estimation
Reid and Dean [12]	2006	596 ^a^	Southern Africa, Northern Europe, and North America	Incisor, Canine and Molar	Histological	Age Estimation
Tan, Sim and Hsu [18]	2017	69	Singapore and Heilongjiang	Permanent Teeth	Histological	Age and Sex Estimation

^a^ Only samples from contemporary humans were considered, although the study included other human groups in the analysis

The specific procedures employed for the analysis of Retzius striae in each study are presented in Table 4. This summary facilitates a clearer and more critical interpretation of the available evidence. Accordingly, all studies prepared samples through histological sectioning with teeth longitudinal cuts. The microscopy method used in all studies was transmitted light microscopy. Five of the seven studies analyzed the internal enamel in the lateral enamel portion. To obtain Retzius periodicity, all studies employed the technique of direct counting of daily cross-striations between adjacent Retzius striae. The studies by Mahoney et al. (2020) [13], Mahoney et al. (2022) [17], McFarlane et al. (2021) [8], McFarlane, Littleton, and Floyd (2014) [16], and Reid and Dean (2006) [12] utilized indirect counting techniques when the striae could not be clearly observed.

**Table 4:** Processing and analysis techniques of Retzius striae

Authors	Sample preparation	Microscopy technique	Tooth area analyzed	Technique for measuring Retzius periodicity
Karaaslan et al. [19]	Longitudinal section; histological sections of 80–100 μm	Transmitted light microscopy (Leica DM5000 B)	Internal enamel, lateral enamel region^a^	Direct counting of daily increments between adjacent Retzius striae.
Mahoney et al. [13]	Longitudinal section; histological sections of 60–90 μm	Transmitted light microscopy (Olympus^®^ BX53)	Internal enamel, lateral enamel region^a^	(1) Direct counting of daily increments between adjacent Retzius striae. (2) Indirect calculation using the enamel secretion rate divided by prism length.
Mahoney et al. [17]	Longitudinal section; thickness adjusted until incremental lines were visible	Transmitted light microscopy (Olympus^®^ BX53)	Internal enamel, lateral enamel region^a^	(1) Direct counting of daily increments between adjacent Retzius striae. (2) Indirect calculation using the enamel secretion rate divided by prism length.
McFarlane et al. [8]	Longitudinal section; histological sections of 100 μm	Transmitted light microscopy with polarization (Olympus BX51)	Internal enamel, lateral enamel region^a^	(1) Direct counting of daily increments between adjacent Retzius striae. (2) Indirect calculation of mean striae spacing divided by the local mean spacing between cross-striations.
McFarlane, Littleton and Floyd [16]	Longitudinal section; histological sections of 100 μm	Transmitted light microscopy (Leica MDR)	(1) External enamel on the buccal surface using high-definition replicas (2) Internal enamel, entire tooth length in the midline area	(1) Direct counting of daily increments between adjacent Retzius striae. (2) Indirect calculation by dividing the distance between two Retzius striae by the mean length of daily striations.
Reid and Dean [12]	Longitudinal section; histological sections of 60–80 μm	Transmitted light microscopy with polarization (Zeiss Universal)	Internal enamel, cervical buccal region of anterior teeth; mesiolingual or mesiobuccal region of posterior teeth.	(1) Direct counting of daily increments between adjacent Retzius striae. (2) Indirect calculation by dividing the distance between adjacent long-period markings by the distance between cross-striations.
Tan, Sim and Hsu [18]	Longitudinal section; histological sections of 80–100 μm	Transmitted light microscopy with polarization (Olympus BX51)	Internal enamel, lateral enamel region^a^	Direct counting of daily increments between adjacent Retzius striae.

^a^ The analysis of Retzius striae periodicity is performed in the internal enamel on histological sections. The external surface, especially the lateral enamel, is used only to select areas where the internal striae appear more clearly or where they correspond to perikymata

Supplementary Table S1 presents the Retzius periodicity values (minimum, mean, maximum, and mode), in days, reported by the included studies. This table allows for direct comparison across the examined teeth and populations. Studies that investigated potential differences associated with biological sex or population variability are also identified, ensuring accurate interpretation of the results and facilitating assessment of the applicability of these findings in Forensic Anthropology. Retzius periodicity in the included studies ranged from 5 to 12 days. The studies by Karaaslan et al. (2021) [19] and Tan, Sim, and Hsu (2017) [18] evaluated permanent teeth but did not specify which teeth were assessed. Mahoney et al. (2022) [17] evaluated deciduous molars. Mahoney et al. (2020) [13], Mahoney et al. (2022) [17], and Tan, Sim, and Hsu (2017) [18] (in the Singapore sample) assessed differences in Retzius periodicity between males and females.

### Methodological quality and risk of bias assessment

In order to assess the methodological quality and risk of bias of the included studies, the Checklist for Analytical Cross-Sectional Studies, proposed by the JBI, was used [27]. Through this checklist, it was possible to determine that all studies were classified as having a low risk of bias (Table 5).

**Table 5:** Methodological quality and risk of bias assessment of the studies according to the Checklist for Analytical Cross-Sectional Studies proposed by The Joanna Briggs Institute [27]. The checklist consists of eight questions (Q), each allowing the following possible answers: “Yes”, “No”, “Unclear” (U), or “Not Applicable” (NA). The questions are: (1) Were the criteria for inclusion in the sample clearly defined? (2) Were the study subjects and the setting described in detail? (3) Was the exposure measured in a valid and reliable way? (4) Were objective, standard criteria used for measurement of the condition? (5) Were confounding factors identified? (6) Were strategies to deal with confounding factors stated? (7) Were the outcomes measured in a valid and reliable way? (8) Was appropriate statistical analysis used?

Study	Q1	Q2	Q3	Q4	Q5	Q6	Q7	Q8	Percentage of “Yes”	Risk of bias
Karaaslan et al. [19]	Yes	Yes	Yes	Yes	Yes	Yes	Yes	Yes	100%	Low
Mahoney et al. [13]	Yes	Yes	Yes	Yes	Yes	Yes	U	Yes	87.5%	Low
Mahoney et al. [17]	Yes	U	Yes	Yes	Yes	Yes	U	Yes	75%	Low
McFarlane et al. [8]	Yes	Yes	Yes	Yes	Yes	Yes	U	Yes	87.5%	Low
McFarlane, Littleton and Floyd [16]	Yes	Yes	U	Yes	Yes	Yes	U	Yes	75%	Low
Reid and Dean [12]	Yes	Yes	U	Yes	NA	NA	U	Yes	75%	Low
Tan, Sim and Hsu [18]	Yes	Yes	Yes	Yes	Yes	Yes	U	Yes	87.5%	Low

## Discussion

Retzius lines represent microstructural records of dental enamel, observed as incremental markings [9]. These features represent periodic patterns of enamel deposition and have been extensively investigated due to their relevance for understanding the dynamics and variability of human dental development [8]. Consequently, they provide an important foundation for research in biological anthropology and related fields. In this context, the present systematic literature review analyzed the available evidence to assess the applicability of Retzius lines in forensic anthropology.

As shown in Fig. 1, a comprehensive literature search was conducted, which enabled the identification of seven studies addressing Retzius lines in contemporary humans. Overall, these studies evaluated the relationship between Retzius line periodicity and biological characteristics of interest in forensic anthropology.

Through qualitative analysis, as shown in Table 5, all studies presented a low risk of bias, indicating high methodological quality. The study by Reid and Dean (2006) [12] included two “Not Applicable” responses, which were not considered in the calculation of the relative (%) values of positive responses. This is explained by the fact that the study employed a descriptive design and did not involve causal comparisons between groups; therefore, the identification and control of potential confounding factors do not fall within the methodological approach adopted by the authors.

All studies assessed Retzius lines using histological methods, as these are microscopic structures present in dental enamel. As shown in Table 4, it can be observed that in all studies Retzius lines were examined microscopically under transmitted light and most studies examined enamel in the lateral region, precisely because it is an area where the distinction between Retzius lines and accentuated or pathological lines is clearer, since the Retzius lines can be traced all the way to the enamel surface [8].

Histological methods are considered destructive and time-consuming when compared to methods that analyze dental development or radiographic techniques, and, moreover, the available sample sizes are generally small [12]. However, there is potential for a greater understanding of the various stages of dental formation, which are difficult to define and observe in radiographs but are of fundamental importance in comparative studies [12].

An alternative to histological methods for assessing Retzius lines, described by McFarlane, Littleton, and Floyd (2014) [16], is the external examination of perikymata on the enamel surface, which is a non-destructive method. Theoretically, the number of perikymata would reflect the number of regular Retzius lines observed in the imbricated enamel; however, in practice, these counts may differ slightly [15]. In their study, a strong correlation was identified between the total number of lines and periodicity in the mandibular canines analyzed [16]. According to the authors, certain regions of the tooth are more reliable for perikymata counts, such as the cervical Half [16]. Additional analyses in other teeth and populations are necessary to use this alternative reliably [16].

As shown in Table 3, the studies assessed Retzius lines using samples composed of different tooth types. In the past, it was believed that Retzius line periodicity was the same across all teeth of a single individual [28]. FitzGerald (1998) [28] directly evaluated this premise and found that the periodicity of Retzius lines remained constant among the anterior teeth of the same individual. However, although this assumption has guided several investigations in anthropological contexts, the existence of intraindividual variation between different tooth types may compromise analyses that assume a single Retzius line periodicity to be representative of the individual [8].

In this regard, some more recent studies have concluded that periodicity can differ between teeth [8, 12, 13], generally being higher in anterior teeth and lower in posterior teeth [10, 12], as observed in the results presented in Supplementary Table S1. Therefore, transferring the periodicity from one tooth to another may result in significant errors in estimates of enamel growth and age at death, reaching discrepancies of up to one year [8]. One possible explanation is related to tooth anatomy and microstructure, which may potentially influence periodicity [8].

In addition, based on Table 3, it can be observed that the studies evaluated some potential applications of Retzius lines in forensic anthropology, including their use for estimating age, sex, and stature. Regarding age estimation, the chronological nature of enamel formation allows for the determination of an individual’s age at various stages of crown development [16]. This aspect of dental development can enable accurate assessments of age at death in subadults [8, 16, 18].

The study by Reid and Dean (2006) [12] shows that Retzius lines are a useful marker for estimating age at death in subadults, as they allow for an accurate reconstruction of the total enamel formation time. However, the method requires caution, since both Retzius line periodicity and the timing of mineralization onset vary between teeth and among individuals [12]. When these variables are accurately measured histologically, the analysis can outperform radiographic methods and be suitable for forensic applications, including cases in which only dental fragments remain [12].

Tan et al. (2017) [18] demonstrated that knowledge of the average Retzius line periodicity values in the Chinese populations of Heilongjiang and Singapore may allow for more accurate age estimations in forensic odontology based on deciduous and still-developing permanent teeth.

Sex estimation was addressed by the study of Mahoney et al. (2020) [13], which analyzed third molars and indicated that Retzius line periodicity in these teeth may be associated with sexual dimorphism. According to the authors, females exhibit higher periodicity compared to males [13]. However, there was a considerable degree of overlap between the sexes in the Retzius line periodicity data. Mahoney et al. (2022) [17] reported similar results when evaluating Retzius line periodicity in deciduous molars. In contrast, Tan et al. (2017) [18] found no statistically significant difference between sexes based on Retzius line periodicity, assessing primarily premolars and molars. Future studies could benefit from investigating sexual dimorphism of Retzius lines in canines, which are considered the most dimorphic teeth [18].

For stature estimation, the study by Karaaslan et al. (2020) [19] reported a negative correlation between Retzius line periodicity and stature when analyzing permanent teeth in a large intraspecific sample of individuals from Beijing and Lhasa; that is, taller individuals tended to exhibit lower Retzius line periodicity. Mahoney et al. (2022) [17] assessed, through regression analyses, that Retzius line periodicity in deciduous molars was not significantly related to total height gains during adolescence.

Additionally, although weight and body mass are not traditionally included in the biological profile used in forensic anthropology, the studies by Mahoney et al. (2022) [17] and Karaaslan et al. (2020) [19] evaluated whether the biological rhythm associated with Retzius line periodicity is related to weight or body mass. Mahoney et al. (2022) [17] assessed Retzius line periodicity in deciduous molars and compared these values with weight and mass gains in adolescents over 14 months. Those with a faster periodicity, of 5 to 6 days, gained less weight and mass [17]. Conversely, individuals with a slower periodicity, of 7 to 8 days, were more likely to gain weight and mass [17]. Karaaslan et al. (2020) [19] demonstrated that Retzius line periodicity, when assessed in permanent teeth, is correlated with weight; however, only a weak correlation was found.

However, these findings should also be interpreted in light of the methodological and interpretative limitations reported by the reviewed studies. Many of the observed associations are still based on relatively small samples or specific population groups and, therefore, should be interpreted with caution [17, 19, 21]. Another important limitation concerns the methodological difficulty involved in the visualization and counting of Retzius lines, since factors such as dental wear, carious lesions, enamel cracks, and accentuated striae may interfere with periodicity assessment and contribute to intra- and interobserver variability [18, 19]. Furthermore, histological preparation and microscopic interpretation remain technically complex procedures that require specialized training and standardized protocols to ensure reliable and reproducible analyses [12, 18, 19].

In this context, the analysis of Retzius lines presents promising perspectives for forensic anthropology; however, the scarcity of studies conducted in contemporary populations still limits the establishment of robust conclusions regarding their applicability. The reviewed studies highlighted the need for future investigations involving larger, methodologically standardized, and population-representative samples in order to improve the reproducibility, reliability, and forensic validity of Retzius line analyses [12, 13, 17, 18]. In addition, the authors emphasized the importance of standardizing protocols related to sample collection, histological preparation, and microscopic analyses, as well as incorporating complementary methodological approaches, such as laser scanning confocal microscopy, to reduce potential measurement errors and improve the accuracy of histological assessments [18].

## Conclusion

In forensic anthropology, the applicability of Retzius lines has been explored for the estimation of age, sex, and stature. The available findings indicate that they constitute a useful marker for age-at-death estimation in children and subadults, as they allow for the accurate reconstruction of the total enamel formation time. Regarding sex estimation, recent literature suggests the existence of sexual dimorphism in Retzius line periodicity; however, the magnitude of this dimorphism appears to depend on tooth type and the population studied, indicating that further investigations are necessary to confirm its forensic utility. Concerning stature, although one study suggested a possible correlation between periodicity and skeletal growth, primary evidence remains insufficient to support practical applications. Furthermore, although weight and body mass are not traditionally included in the biological profile in forensic anthropology, studies have explored associations between Retzius line periodicity and these variables; nevertheless, such findings are still preliminary and do not support forensic applicability. Thus, the need for additional methodologically standardized primary studies with representative samples is evident to assess the reliability and validity of these applications safely in a forensic context.

## Supplementary information

Below is the link to the electronic supplementary material.

Supplementary Material 1

## Data Availability

Data sharing not applicable to this article as no datasets were generated or analysed during the current study.
