# Supplementary material for: Applicability of Retzius striae in forensic anthropology: a systematic review
Source: Int J Legal Med. 2026 Aug 13;140(6):3623–33. doi: 10.1007/s00414-026-03954-3 (PMC13658353; doi:10.1007/s00414-026-03954-3)
Supplement: Supplementary file 1 [file 414_2026_3954_MOESM1_ESM.docx]

**INTERNATIONAL JOURNAL OF LEGAL MEDICINE**

**Applicability of Retzius Striae in Forensic Anthropology: A Systematic Review**

Clarice de Araújo OLIVEIRA^1^, Antônio do Rêgo CASTELO-BRANCO^1^, Paulo Henrique Viana PINTO^1,2^, Ricardo Henrique Alves da SILVA^3*^

^1^Department of Pathology and Legal Medicine, Ribeirão Preto Medical School, University of São Paulo (USP). Ribeirão Preto, São Paulo, Brazil.

²Forensic Odontologist, Bahia State Scientific Police, Bahia, Brazil.

^3^Department of Stomatology, Public Health, and Forensic Odontology, Ribeirão Preto School of Dentistry, University of São Paulo (USP). Ribeirão Preto, São Paulo, Brazil.

* **CORRESPONDING AUTHOR’S ADDRESS**

Ricardo Henrique Alves da Silva

[ricardohenrique@usp.br](mailto:ricardohenrique@usp.br)

**Supplementary Table S1** Retzius periodicity assessed in each study.

| **Authors** | **Sample Origin** | **Tooth** | **Sex** | **Retzius Periodicity (in days)** | | | |
| --- | --- | --- | --- | --- | --- | --- | --- |
|  |  |  |  | **Minimum** | **Mean** | **Maximum** | **Mode** |
| Karaaslan *et al*. [19] | Beijing | Permanent Teeth | Both sexes | 5 | 7.47 | 10 | 8 |
|  | Lhasa | Permanent Teeth | Both sexes | 5 | 7.69 | 11 | 8 |
| Mahoney *et al*. [13] | Northern Great Britain | Third Molars | Both sexes | 6 | 8.15 | 11 | 8 |
|  |  |  | Male | 6 | 7.65 | 10 | 7 |
|  |  |  | Female | 7 | 8.63 | 11 | 8 |
| Mahoney *et al*. [17] | New Zealand | Deciduous Molars | Both sexes | 5 | 7.26 | 10 | 6 |
|  |  |  | Male | 5 | 6.96 | 10 | 6 |
|  |  |  | Female | 5 | 7.50 | 10 | 8 |
| McFarlane *et al*. [8] | United Kingdom | Incisor | Both sexes | 9 | 9.91 | 11 | 9, 10 |
|  |  | Canine | Both sexes | 7 | 9.93 | 12 | 11 |
|  |  | Premolar | Both sexes | 7 | 8.54 | 10 | 8 |
|  |  | Molar | Both sexes | 7 | 8.42 | 11 | 8 |
|  | Southern Africa | Incisor | Both sexes | 8 | 9.90 | 12 | 9 |
|  |  | Canine | Both sexes | 8 | 9.55 | 12 | 9 |
|  |  | Premolar | Both sexes | 7 | 9.08 | 12 | 9 |
|  |  | Molar | Both sexes | 6 | 8.26 | 11 | 8, 9 |
| McFarlane, Littleton and Floyd [16] | New Zealand | Canine | Both sexes | 7 | 8.50 | 10 | 8 |
| Reid and Dean [12] | Southern Africa | Maxillary Central Incisor | Both sexes | 8 | 9.60 | 12 | 10 |
|  |  | Maxillary Lateral Incisor | Both sexes | 7 | 9.55 | 11 | 10 |
|  |  | Maxillary Canine | Both sexes | 6 | 8.90 | 12 | 9 |
|  |  | Mandibular Central Incisor | Both sexes | 8 | 8.97 | 11 | 9 |
|  |  | Mandibular Lateral Incisor | Both sexes | 7 | 9.26 | 12 | 9 |
|  |  | Mandibular Canine | Both sexes | 7 | 8.54 | 11 | 8 |
|  |  | Maxillary First Molar – Mesial Palatal Cusp | Both sexes | 6 | 8.78 | 12 | 8 |
|  |  | Maxillary First Molar – Mesial Buccal Cusp | Both sexes | 6 | 8.73 | 12 | 8 |
|  |  | Maxillary Second Molar – Mesial Palatal Cusp | Both sexes | 7 | 8.61 | 11 | 8 |
|  |  | Maxillary Second Molar – Mesial Buccal Cusp | Both sexes | 7 | 8.58 | 11 | 8 |
|  |  | Maxillary Third Molar – Mesial Palatal Cusp | Both sexes | 6 | 8.30 | 10 | 8 |
|  |  | Maxillary Third Molar – Mesial Buccal Cusp | Both sexes | 6 | 8.26 | 10 | 8 |
|  |  | Mandibular First Molar – Mesial Lingual Cusp | Both sexes | 7 | 8.59 | 10 | 9 |
|  |  | Mandibular First Molar – Mesial Buccal Cusp | Both sexes | 7 | 8.57 | 10 | 9 |
|  |  | Mandibular Second Molar – Mesial Lingual Cusp | Both sexes | 7 | 8.70 | 10 | 8 |
|  |  | Mandibular Second Molar – Mesial Buccal Cusp | Both sexes | 7 | 9.00 | 12 | 8 |
|  |  | Mandibular Third Molar – Mesial Lingual Cusp | Both sexes | 7 | 8.21 | 9 | 8 |
|  |  | Mandibular Third Molar – Mesial Buccal Cusp | Both sexes | 7 | 8.14 | 9 | 8 |
|  | Northern Europe | Maxillary Central Incisor | Both sexes | 7 | 8.47 | 10 | 8 |
|  |  | Maxillary Lateral Incisor | Both sexes | 8 | 9.13 | 11 | 9 |
|  |  | Maxillary Canine | Both sexes | 8 | 9.00 | 11 | 9 |
|  |  | Mandibular Central Incisor | Both sexes | 7 | 8.71 | 9 | 8 |
|  |  | Mandibular Lateral Incisor | Both sexes | 7 | 8.85 | 11 | 9 |
|  |  | Mandibular Canine | Both sexes | 7 | 8.69 | 10 | 8 |
|  |  | Maxillary First Molar – Mesial Palatal Cusp | Both sexes | 6 | 8.27 | 10 | 8 |
|  |  | Maxillary First Molar – Mesial Buccal Cusp | Both sexes | 6 | 8.27 | 10 | 8 |
|  |  | Maxillary Second Molar – Mesial Palatal Cusp | Both sexes | 6 | 7.73 | 9 | 8 |
|  |  | Maxillary Second Molar – Mesial Buccal Cusp | Both sexes | 6 | 7.69 | 9 | 8 |
|  |  | Maxillary Third Molar – Mesial Palatal Cusp | Both sexes | 6 | 7.94 | 9 | 8 |
|  |  | Maxillary Third Molar – Mesial Buccal Cusp | Both sexes | 6 | 7.94 | 9 | 8 |
|  |  | Mandibular First Molar – Mesial Lingual Cusp | Both sexes | 7 | 8.47 | 11 | 8 |
|  |  | Mandibular First Molar – Mesial Buccal Cusp | Both sexes | 7 | 8.47 | 11 | 8 |
|  |  | Mandibular Second Molar – Mesial Lingual Cusp | Both sexes | 7 | 8.31 | 9 | 8 |
|  |  | Mandibular Second Molar – Mesial Buccal Cusp | Both sexes | 7 | 8.31 | 9 | 8 |
|  |  | Mandibular Third Molar – Mesial Lingual Cusp | Both sexes | 6 | 7.73 | 10 | 8 |
|  |  | Mandibular Third Molar – Mesial Buccal Cusp | Both sexes | 6 | 7.73 | 10 | 8 |
|  | North America – Caucasian | Maxillary Third Molar – Mesial Palatal Cusp | Both sexes | 7 | 7.78 | 9 | 8 |
|  |  | Maxillary Third Molar – Mesial Buccal Cusp | Both sexes | 7 | 7.75 | 9 | 8 |
|  |  | Mandibular Third Molar – Mesial Lingual Cusp | Both sexes | 7 | 7.86 | 9 | 8 |
|  |  | Mandibular Third Molar – Mesial Buccal Cusp | Both sexes | 7 | 7.86 | 9 | 8 |
|  | North America – Mixed | Maxillary Third Molar – Mesial Palatal Cusp | Both sexes | 7 | 7.88 | 9 | 8 |
|  |  | Maxillary Third Molar – Mesial Buccal Cusp | Both sexes | 7 | 7.86 | 9 | 8 |
|  |  | Mandibular Third Molar – Mesial Lingual Cusp | Both sexes | 7 | 8.00 | 9 | 8 |
|  |  | Mandibular Third Molar – Mesial Buccal Cusp | Both sexes | 7 | 7.92 | 9 | 8 |
| Tan, Sim and Hsu [18] | Singapore | Permanent Teeth | Both sexes | 6 | 7.25 | 8 | 7 |
|  |  |  | Male | 6 | 7.27 | 8 | 7 |
|  |  |  | Female | 7 | 7.30 | 8 | 7 |
|  | Heilongjiang | Permanent Teeth | Both sexes | 6 | 6.60 | 8 | 6 |
